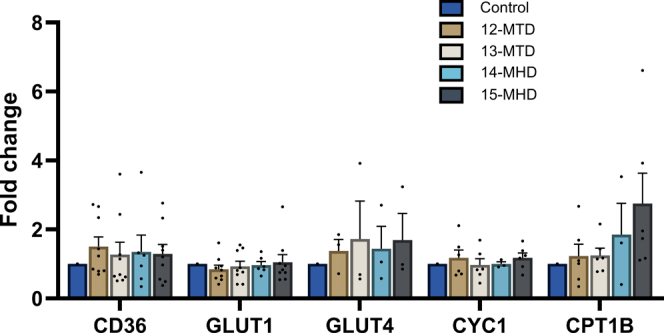# Divergent effects of monomethyl branched-chain fatty acids on energy metabolism and insulin signaling in human myotubes

**DOI:** 10.1016/j.jlr.2025.100764

**Published:** 2025-02-24

**Authors:** Parmeshwar Bajirao Katare, Ragna H. Tingstad, Sivar T. Beajani, Jørgen Pasjkurov Indseth, Vibeke H. Telle-Hansen, Mari C.W. Myhrstad, Arild C. Rustan, Lars Eide, Oliwia Witczak, Vigdis Aas

**Affiliations:** 1 Section for Pharmacology and Pharmaceutical Biosciences, Department of Pharmacy, University of Oslo, Oslo, Norway; 2 Department of Life Sciences and Health, Oslo Metropolitan University – OsloMet, Oslo, Norway; 3 Department of Nursing and Health Promotion, Oslo Metropolitan University – OsloMet, Oslo, Norway; 4 Department of Medical Biochemistry, Institute of Clinical Medicine, University of Oslo, Oslo, Norway

**Keywords:** energy homeostasis, obesity and metabolic diseases, skeletal muscle metabolism, dairy products

## Abstract

Branched-chain fatty acids (BCFAs) are predominantly saturated fatty acids with one or more methyl branches on the carbon chain, typically found in dairy products and measured in micromolar concentrations in human plasma. The biological function of BCFAs in humans remains ill-defined, but a relationship between circulating BCFAs and cardiometabolic health has been suggested. The objective of this study was to evaluate the impact of BCFAs on energy metabolism in human myotubes. The results revealed distinct effects of BCFAs. 12-Methyltetradecanoic acid (12-MTD) increased glucose uptake and glycogen synthesis, while 13-methyltetradecanoic acid (13-MTD), 14-methylhexadecanoic acid (14-MHD), and 15-methylhexadecanoic acid (15-MHD) increased oleic acid uptake and 13-MTD and 15-MHD oleic acid oxidation, indicating a more general stimulatory effect on fatty acid than glucose metabolism. Interestingly, the same BCFAs, 13-MTD, 14-MHD, and 15-MHD, appeared to reduce insulin-stimulated glycogen synthesis. Insulin-stimulated phosphorylation of IRS1 was not apparent after exposure to 12-MTD, 13-MTD, and 15-MHD, whereas insulin-stimulated phosphorylation of Akt was unchanged by BCFAs. Incorporation of [^14^C]leucine into lipids was affected, as 13-MTD increased the total lipid content, and 12-MTD altered the distribution of lipid classes. Metabolic flux analysis indicated that 14-MHD stimulated extracellular acidification. The effects of BCFAs might involve increased mRNA expression of pyruvate dehydrogenase kinase 4. In conclusion, the study demonstrates that different BCFAs have distinct effects on energy metabolism in myotubes, 12-MTD mainly affect glucose metabolism, while 13-MTD, 14-MHD, and 15-MHD modulated oleic acid metabolism. These data suggest that some BCFAs might have therapeutic applications by improving energy metabolism.

Cardiovascular and metabolic disorders, such as type 2 diabetes, are of major concern to public health. According to the International Diabetes Federation, about 10% of adults have diabetes, and cardiovascular diseases are still the leading cause of death globally (WHO). Primary prevention of these disorders by lifestyle interventions is therefore an important goal. Consumption of dairy products has been associated with negative health effects due to the high content of saturated fatty acids, resulting in increased blood cholesterol and increased risk of type 2 diabetes (1, 2). However, the unhealthy effect of dairy products is debated (3, 4, 5). A meta-analysis published in 2019 revealed that the association between dairy intake and cardiovascular disease is complex and probably favorable (6). Studies suggest that the content of branched-chain fatty acids (BCFAs) in human plasma may contribute to positive health effects related to the consumption of dairy products (7, 8, 9, 10).

BCFAs are a group of mostly saturated and long-chain fatty acids with one or more methyl groups attached to the carbon chain. Based on branch point position, the monomethyl BCFAs are distinguished as *iso*-BCFA with the methyl group at n-2 and *anteiso*-BCFA with the methyl group at n-3 (11). BCFAs are major components of the vernix caseosa and also present in newborn gastrointestinal tract (12). Further, they have been detected in various other human tissues and fluids, including adipose tissue, breast milk, colon, and serum (9). The BCFAs are synthesized in cytosol from intermediates from the mitochondrial degradation of branched-chain amino acids (BCAAs), leucine, isoleucine, and valine (9). The concentration of BCFAs has been measured in the range 2–12 μM in human plasma, and the plasma concentrations were sensitive to overnight fasting, suggesting that both diet and endogenous synthesis contribute to the circulating levels (13). Dairy products serve as a predominant dietary source (9). Abundant BCFAs in milk are the *anteiso*-BCFAs 12-MTD (12-methyltetradecanoic acid) and 14-MHD (14-methylhexadecanoic acid), and the *iso*-BCFA 15-MHD (15-methylhexadecanoic acid) (14). Other dietary sources of BCFAs are ruminant food products and fermented vegetables (9). The human gut microbiota might also contribute to plasma BCFAs by supplying branched-chain keto acids, the precursors of de novo BCFA synthesis (15, 16).

The role of BCFAs as energy source and/or metabolic regulators in humans is probably limited due to low concentrations. Still, a few studies have looked at the association between BCFAs and certain disorders (10), and there is growing evidence that BCFAs are associated with metabolic diseases and inflammation (17, 18) and can have important roles in energy homeostasis (19). Interestingly, subjects with excess body weight have a lower fraction of *iso*-BCFAs in serum than lean controls (18), and the fraction of BCFAs in serum of morbidly obese subjects increased to a level not different from lean controls after gastric bypass (20). A prospective case control study showed that consumption of dairy fat (rich in BCFAs) was negatively associated with cardiovascular risk factors (21). Furthermore, serum levels of *iso*-BCFAs were found to be negatively correlated with C-reactive protein concentration, hypertriglyceridemia, and insulin concentration in individuals with obesity (18).

Skeletal muscle is a major organ for oxidation of fatty acids and carbohydrates and contributes significantly to peripheral insulin resistance in type 2 diabetes (22); therefore, it is important to understand the effect of BCFAs on skeletal muscle. Skeletal muscle is also the main site of metabolism of BCAAs. The skeletal muscle metabolism of BCAAs is impaired in type 2 diabetes (23, 24), and obesity and type 2 diabetes are both associated with increased plasma levels of BCAAs (23). The source of increased plasma BCAAs is not known, nor whether the metabolism of BCAAs and BCFAs is connected in humans. Nevertheless, the observation that plasma BCAA is elevated, while BCFA is reduced in obesity and type 2 diabetes is interesting and might be relevant. We lack information about the accurate role of these less abundant fatty acids in human biological processes, including energy metabolism and cellular signaling in skeletal muscle. Therefore, this study aimed to investigate the effect of BCFAs on energy metabolism and insulin responsiveness in myotubes derived from healthy individuals. Glucose, fatty acid, and leucine metabolism were examined.

## Material and methods

### Materials

Corning® CellBIND® tissue culture plates were from Corning (Schiphol-Rijk, the Netherlands). Dulbecco’s Modified Eagle’s Medium (DMEM) with GlutaMAX™ low glucose, Dulbecco’s phosphate buffered saline (DPBS; with Ca^2+^ and Mg^2+^), heat-inactivated fetal bovine serum (FBS), gentamicin (50 mg/ml), penicillin-streptomycin (10,000 IE/ml), amphotericin B, human epidermal growth factor, trypsin-EDTA, Pierce™ BCA Protein Assay Kit, MicroAmp® Optical 96-well Reaction Plate, MicroAmp® Optical Adhesive Film, Applied Biosystem High-Capacity cDNA Reverse Trascription kit, and primers for real time qPCR were purchased from Thermo Fisher Scientific (Waltham, MA). Insulin (Actrapid® Penfill® 100IE/ml) was from NovoNordisk (Bagsvaerd, Denmark). D-[^14^C(U)]glucose (3.0 mCi/mmol), [1–^14^C]oleic acid (OA, 59.0 mCi/mmol), and L-[^14^C(U)]leucine (59.0 mCi/mmol) were from PerkinElmer NEN® (Boston, MA). Ultima Gold™ XR, Pico Prias 6 ml PE vials, 96-well Isoplate®, UniFilter®-96 GF/B microplates, and TopSeal®-A transparent film was obtained from PerkinElmer (Shelton, CT). 4-(2-hydroxyethyl)-1-piperazineethanesulfonic acid (Hepes), β-mercaptoethanol, dimethyl sulfoxide (DMSO), bovine serum albumin (BSA), dexamethasone, gentamicin, L-glutamine, L-carnitine, protease inhibitor, phosphatase II inhibitor, radioimmunoprecipitation assay buffer, trypan blue 0.4% solution D-glucose, and palmitic acid (16:0) were obtained from Sigma-Aldrich (St. Louis, MO). QIAshredder, QuantiNova SYBR® Green RT-PCR kit, and RNeasy Mini Kit were from QIAGEN (Venlo, the Netherlands).

## Ethics statement

Human skeletal muscle biopsies were obtained after informed written consent and approval by the Regional Committee for Medical and Health Research Ethics South-East, Oslo, Norway (reference number: REK11959). The study was conducted in accordance with the guidelines of the Declaration of Helsinki. All data are pseudonymous, and donor identities are unknown to the authors.

## Methods

### Donor characteristics

Cultured myotubes were derived from biopsies collected from a cohort of eight healthy adult donors, aged 51 ± 4 years, consisting of four males and four females. The donors had a body mass index of 29 ± 2 kg/m^2^ and fasting plasma glucose of 4.2 ± 0.2 mM.

### Cell culture

Human satellite cells were isolated from skeletal muscle biopsy samples from *musculus vastus lateralis*, as previously described (25, 26, 27). In brief, satellite cells were extracted from muscle biopsies, decontaminated of fibroblasts, and grown to passages 3–6. The isolated cells were cultured and proliferated in DMEM-GlutaMAX (5.5 mM glucose) supplemented with 10% FBS, Hepes (25 mM), gentamicin (50 ng/ml), penicillin (25 IU), streptomycin (25 μg/ml), amphotericin B (1.25 μg/ml), human epidermal growth factor (10 ng/ml), dexamethasone (0.39 μg/ml), and 0.05% BSA. Differentiation of myoblasts into myotubes was induced at 80%–90% confluence by changing the medium to DMEM-GlutaMAX (5.5 mM glucose) supplemented with 2% FBS, Hepes (25 mM), gentamicin (50 ng/ml), amphotericin B (1.25 μg/ml), and 25 pM insulin. The cells were cultured at 37°C in a humidified atmosphere containing 5% CO_2_, and the medium was changed every 2–3 days. On the sixth day of differentiation, cells were treated with 100 μM of BCFAs (stock dissolved in DMSO) and/or palmitic acid complexed to fatty acid-free BSA at a ratio 2.5/1. Experiments were carried out 7–8 days after the induction of cell differentiation, and all control cells were added DMSO at the same concentration as BCFA-treated cells (vehicle control).

### Substrate oxidation assay

Skeletal muscle cells were cultured in 96-well CellBIND® microplates. The cells were then given D-[^14^C(U)]glucose (0.5 μCi/ml, 200 μM) or [1^−14^C]oleic acid (0.5 μCi/ml, 100 μM) substrate during 4 h CO_2_-trapping, as described previously (28). The glucose substrate was prepared in DPBS supplemented with Hepes (10 mM), whereas the oleic acid substrate was added in DPBS containing Hepes (10 mM), BSA (40 μM), and L-carnitine (1 mM). Following trapping, the ^14^CO_2_ produced by the cells and cell-associated (CA) radioactivity were measured using a 2,450 MicroBeta^2^ liquid scintillation counter (PerkinElmer). Protein concentration in each well was determined with the Bio-Rad protein assay kit to relate the ^14^CO_2_ and CA data to cellular protein content. Complete substrate oxidation was measured as ^14^CO_2,_ and cellular uptake was calculated as ^14^CO_2_ + CA. The results are given as nmol/mg protein.

### Glycogen synthesis

Myotubes were grown in 6-well CellBIND® plates and incubated for 60 min in serum-free DMEM-Glutamax before D-[^14^C(U)]glucose (1.0 μCi/ml) ± insulin (100 nM) was added. After 2 h, the cells were washed with PBS and lysed in 0.5 ml KOH (1 M). Synthesized glycogen was measured as described (29) and given as nmol/mg protein.

### Measurements of oxygen consumption and extracellular acidification

Skeletal muscle cells were plated and cultured on a XF24-well culture plate (Agilent) and incubated with BCFA (100 μM) for 24 h. Before the measurement, the culture medium was removed and replaced by assay medium (Agilent) supplemented with 5.5 mM glucose, 1 mM sodium pyruvate, and 5 mM glutamine for 1 h at 37°C. Using the Seahorse XF24 bioanalyzer (Agilent, Wilmington, DE), the mitochondrial oxygen consumption rate (OCR), which serves as an indicator of mitochondrial respiration (OXPHOS) and extracellular acidification rate (ECAR), which serves as an indicator of glycolysis, were recorded over time (baseline). Sequential injections of 5 μM oligomycin, 3 μM FCCP (carbonyl cyanide 4-(trifluoromethoxy)phenylhydrazone), and 1 μM rotenone were added to examine proton leak, maximal respiration, and nonmitochondrial respiration, respectively. The OCR and ECAR values were related to the total protein content measured by Pierce BCA Protein Assay kit.

### Gene expression analysis

Total RNA from cultured cells was extracted using the QIAGEN RNeasy Mini Kit according to the manufacturer’s instructions. The RNA was reverse-transcribed into cDNA with the Applied Biosystem High-Capacity cDNA Reverse Transcription Kit using Applied Biosystem 2,720 Thermal Cycler (25°C for 10 min, 37°C for 120 min, and 85°C for 5 min). The resulting cDNA was subjected to real time qPCR using Qiagen QuantiNova SYBR® Green RT-PCR kit using a Stratagene MX3000p qPCR cycler [initial activation at 95°C for 2 min, followed by 35 cycles of denaturation (95°C for 5 s) and combined annealing and extension (60°C for 10 s)]. The following human genes were assessed (with the official gene symbol in parenthesis): pyruvate dehydrogenase kinase 4 (*PDK4*), acc.no.: BC040239, carnitine palmitoyltransferase 1B (*CPT1b*), acc.no.: NM_004377, peroxisome proliferator-activated receptor δ (*PPARD*), acc.no.: BC002715, peroxisome proliferator-activated receptor γ (*PPARG*), acc.no.: L40904, platelet glycoprotein 4 (*CD36*), acc.no.: L06850, solute carrier family 2 facilitated glucose transporter member 1 (*SLC2A1*), acc. no.: K03195, solute carrier family 2 facilitated glucose transporter member 4 (*SLC2A4*) acc. no.: M20747, cytochrome 1c (*CYC1*), acc.no.: NM 001916, ribosomal protein lateral stalk subunit P0 (*RPLP0*) acc.no.: M17885, and glyceraldehyde-3-phosphate dehydrogenase (*GAPDH*), acc. no.: NM002046. The transcription levels were normalized to the housekeeping control gene glyceraldehyde-3-phosphate dehydrogenase (*GAPDH*) or ribosomal protein lateral stalk subunit P0 (*RPLP0*). Primer sequences are given in Table 1.

**Table 1:** Primer sequences for gene expression analysis by real-time qPCR

Gene name (Gene symbol)	Accession number	Forward primer (5′-3′)	Reverse primer (5′-3′)
Pyruvate dehydrogenase kinase 4 (*PDK4*)	BC040239	TTT CCA GA CCA ACC AAT TCA CA	TGC CCG CAT TGC ATT CTT A
Carnitine palmitoyltransferase 1B (*CPT1B*)	NM_004377	CAA AAT TCC CTT CCT GCT CCA AC	CGC TTT GGA AAC CAC ATC CG
Peroxisome proliferator-activated receptor δ (*PPARD*)	BC002715	AGC ATC CTC ACC GGC AAA	ATG TCT CGA TGT CGA TGT CGT GGA TCA C
Peroxisome proliferator-activated receptor γ (*PPARG*)	L40904	AGC CTG CGA AAG CCT TTT G	ATT CCA GTG CAT TGA ACT TCA CA
Platelet glycoprotein 4 (*CD36*)	L06850	AGT CAC TGC GAC ATG ATT AAT GGT	CTG CAA TAC CTG GCT TTT CTC AA
Solute carrier family 2, facilitated glucose transporter member 1 (*GLUT1* or *SLC2A1*)	K03195	CAG CAG CCC TAA GGA TCT CTC A	CCG GCT CGG CTG ACA TC
Solute carrier family 2, facilitated glucose transporter member 4 (*GLUT4* or *SLC2A4*)	M20747	ACC CTG GTC CTT GCT GTG TT	ACC CCA ATG TTG TAC CCA AAC T
Cytochrome c1 (CYC1)	NM001916	CTG CCA ACA ACG GAG CAT T	CGT GAG CAG GGA GAA GAC GTA
Glyceraldehyde-3-phosphate dehydrogenase (*GAPDH*)	NM002046	TGC ACC ACC AAC TGC TTA GC	GGC ATG GAC TGT GGT CAT GAG
Acidic ribosomal phosphoprotein P0 (*RPLP0*)	M17885	CCA TTC TAT CAT CAA CGG GTA CAA	AGC AAG TGG GAA GGT GTA ATC C

### Immunoblotting

After exposure to BCFAs (100 μM), myotubes were lysed using radioimmunoprecipitation assay buffer, and the protein concentration was determined by Pierce BCA protein assay. The proteins were electrophoretically separated on 4%–20% Mini-Protean TGX™ gels with Tris/glycine buffer (pH 8.3) (Bio-Rad, Hercules, CA) and transferred to polyvinylidene fluoride membranes. After blocking in TBST with 3% BSA or 5% skimmed milk, the membranes were incubated with the primary antibodies against Akt (1:1,000, #9272, Cell Signaling Technology, Danvers, MA), phospho-Akt (Ser473; 1:1,000, #9271, Cell Signaling Technology, Danvers, MA), IRS-1 (1:400, #06–248, Sigma-Aldrich, St. Louis, MO), phospho-IRS1 (Tyr612; 1:750, #44-816G, Thermo Fisher Scientific, Waltham, MA), and alpha-tubulin (1:1,000, #GR3194994-1, Abcam, Cambridge, UK or 1:3,000, #T6199, Sigma-Aldrich, St. Louis, MO) at 4°C overnight. After washing in TBST, an anti-rabbit HRP-conjugated secondary antibody (#7074) or an anti-mouse HRP-conjugated secondary antibody (# 7076) (both 1:2000, Cell Signaling Technology, Danvers, MA) was added. Bands were visualized by enhanced chemiluminescence reagent (Bio-Rad, Hercules, CA) using Chemidoc XRS + Imager (Bio-Rad, Hercules, CA) and analyzed in Image J (NIH, Bethesda, MD).

### Leucine incorporation into proteins/protein synthesis

Myotubes were cultured in 24-well CellBIND® plates. After 6 days of differentiation, the cells were incubated with BCFA (100 μM) and [^14^C]leucine (1 μCi/ml, 0.8 mM) for 24 h. The cells were then washed with PBS and lysed in 0.01% SDS. The amount of protein per well was determined by Pierce BCA protein assay. Protein from the cell lysates was precipitated with 6% BSA and 50% trichloroacetic acid overnight at −20°C. The next day, the cell lysates were centrifuged to form a protein pellet. The pellet was washed in acetone before being centrifuged, air-dried, and resuspended in SDS-NaOH. Radioactivity was measured by liquid scintillation (Packard Tri-Carb 1900 TR, PerkinElmer), and the amount of labeled protein was normalized to the total cell protein concentration.

### Leucine incorporation into lipids/lipid synthesis

Myotubes were cultured in 6-well CellBIND® plates. After 6 days of differentiation, the cells were incubated with BCFAs (100 μM) and [^14^C]leucine (1 μCi/ml, 0.8 mM) for 24 h. The cells were then washed with PBS and lysed in 0.01% SDS. Lipids were extracted with chloroform: methanol (2:1) as described (30) and separated by thin layer liquid chromatography as described (31). The bands were quantified by liquid scintillation. The lipid content is given as nmol/mg protein.

### Statistical analysis

All values are presented as mean ± SEM unless stated otherwise in the figure legends. The value *n* represents the number of individual experiments (=biological replicates), and each experiment was performed with 2–8 (specified in text) technical replicates. Statistical analysis and graphs were performed using GraphPad Prism 8.0.1 for Windows (GraphPad Software Inc., San Diego, CA). The statistical difference between treated cells and control was analyzed by either paired (unnormalized data) or one sample (normalized data) Student’s *t* test. Statistical differences between treatment groups were examined by either a mixed effects model (restricted maximum likelihood), one-way analysis of variance (ANOVA), or two-way ANOVA, and all were followed by uncorrected Fisher’s least significant difference test to determine differences. *P* < 0.05 was considered significant for all statistical analyses.

## Results

### Impact of BCFAs on uptake and oxidation of glucose and oleic acid

**Fig. 1:**
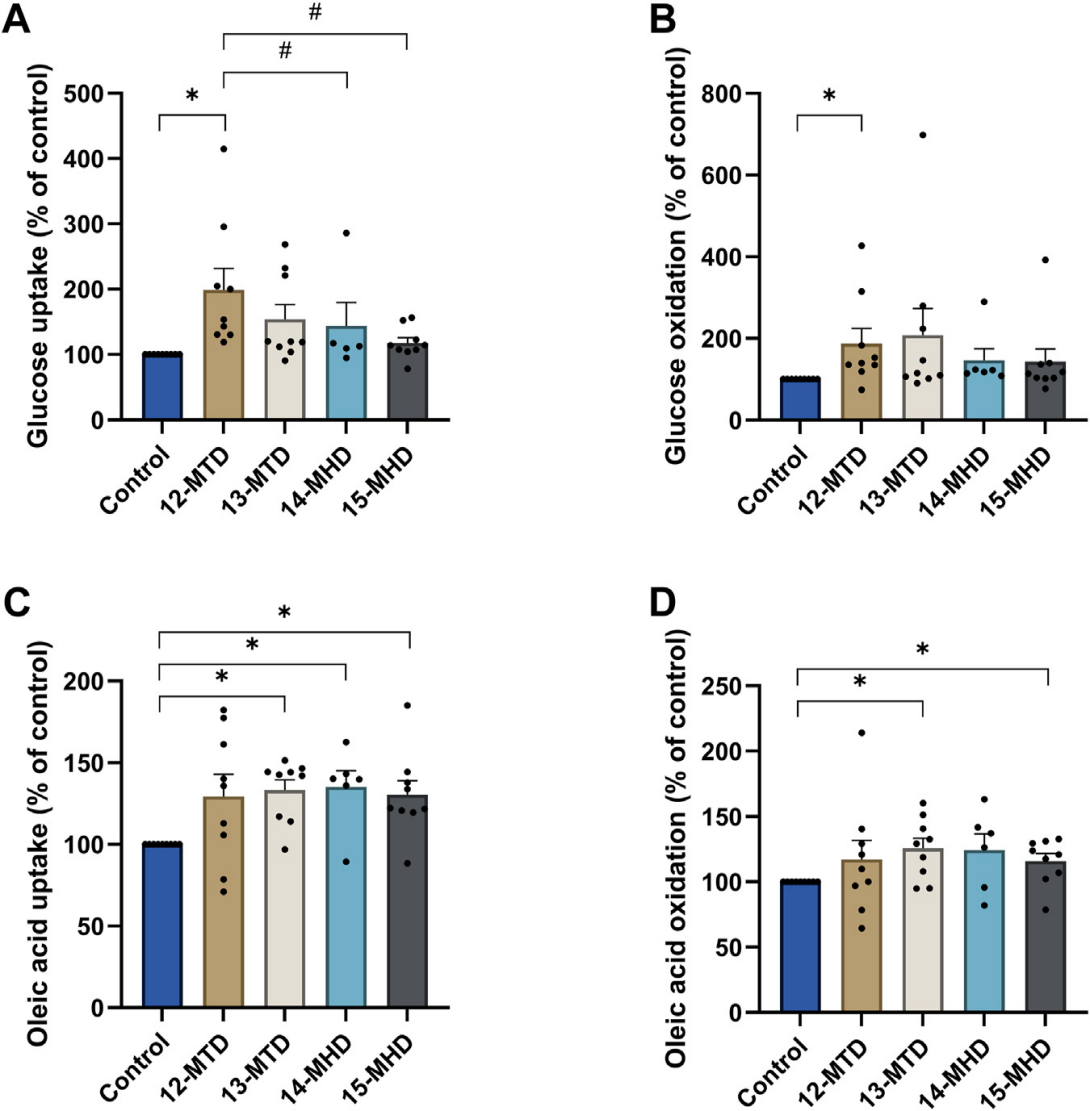
Glucose and oleic acid uptake and oxidation in human myotubes treated with branched-chain fatty acids. Human myotubes were incubated with 100 μM 12-MTD (12-methyltetradecanoic acid), 13-MTD (13-methyltetradecanoic acid), 14-MHD (14-methylhexadecanoic acid), or 15-MHD (15-methylhexadecanoic acid) for 24 h before glucose (A, B) and oleic acid (C, D) metabolism were assessed by addition of a medium containing [^14^C]glucose (0.5 μCi/ml, 200 μM) or [^14^C]oleic acid (0.5 μCi/ml, 100 μM) for 4 h. Cell-associated radioactivity and trapped CO_2_ (oxidation) were measured as described in methods. Uptake of glucose and oleic acid were calculated as the sum of oxidized substrate and cell-associated radioactivity. In control cells, glucose uptake and oxidation were 88.6 ± 28.9 nmol/mg and 63.7 ± 28.9 nmol/mg, respectively, and oleic acid uptake and oxidation 15.5 ± 3.9 nmol/mg and 1.4 ± 0.3 nmol/mg, respectively. Data are given as mean ± SEM from 6–9 individual experiments (n = 6–9, each with 4 technical replicates). ∗Significantly different from control (*P* < 0.05) by one sample Student’s *t* test. #Significantly different with the mixed effects model (REML) followed by uncorrected Fisher’s LSD test (*P* < 0.05).

To investigate whether the monomethyl branched fatty acids possibly could influence energy metabolism in human myotubes, we compared the effect of *anteiso- and iso*-forms of the tetradecanoic (12-MTD and 13-MTD) and the hexadecanoic (14-MHD and 15 MHD) acids by exposing the cells for 24 h prior to measurement of energy metabolism using radiolabeled glucose and oleic acid (Fig. 1). The BCFAs increased glucose metabolism in a BCFA type-specific manner (Fig. 1A, B). The *anteiso*-BCFA (12-MTD) caused a 2-fold increase in glucose uptake and oxidation (Fig. 1A, B), while the others did not significantly affect glucose metabolism. The effect of 12-MTD on glucose uptake was however significantly different from the iso-form hexadecanoic acids 14-MHD and 15-MHD (Fig. 1A).

**Supplemental fig 1:**
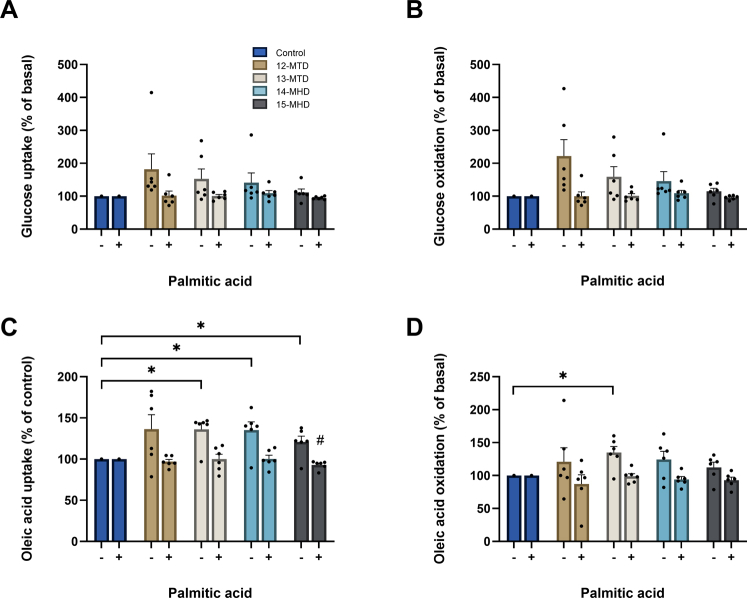


All the BCFAs tended to increase both uptake (Fig. 1C) and oxidation of oleic acid (Fig. 1D), although statistical significance was not reached by 12-MTD [neither uptake (*P* = 0.06) nor oxidation] or 14-MHD (oxidation). Oleic acid uptake was increased by 30%–35% and oxidation by 16%–25%. There were no differences between the individual BCFAs. The cells were also exposed to the BCFAs in combination with palmitic acid to study the effect of BCFAs in metabolically challenged myotubes (supplemental Fig. S1). The copresence of palmitic acid abolished the effects on both glucose (supplemental Fig. S1*A*, *B*) and oleic acid metabolism (supplemental Fig. S1*C*, *D*). These results indicate that various BCFAs affect energy metabolism differently and that the effects of BCFAs are compromised in metabolically disturbed myotubes.

### Mitochondrial function and glycolytic activity after exposure to BCFAs

**Fig. 2:**
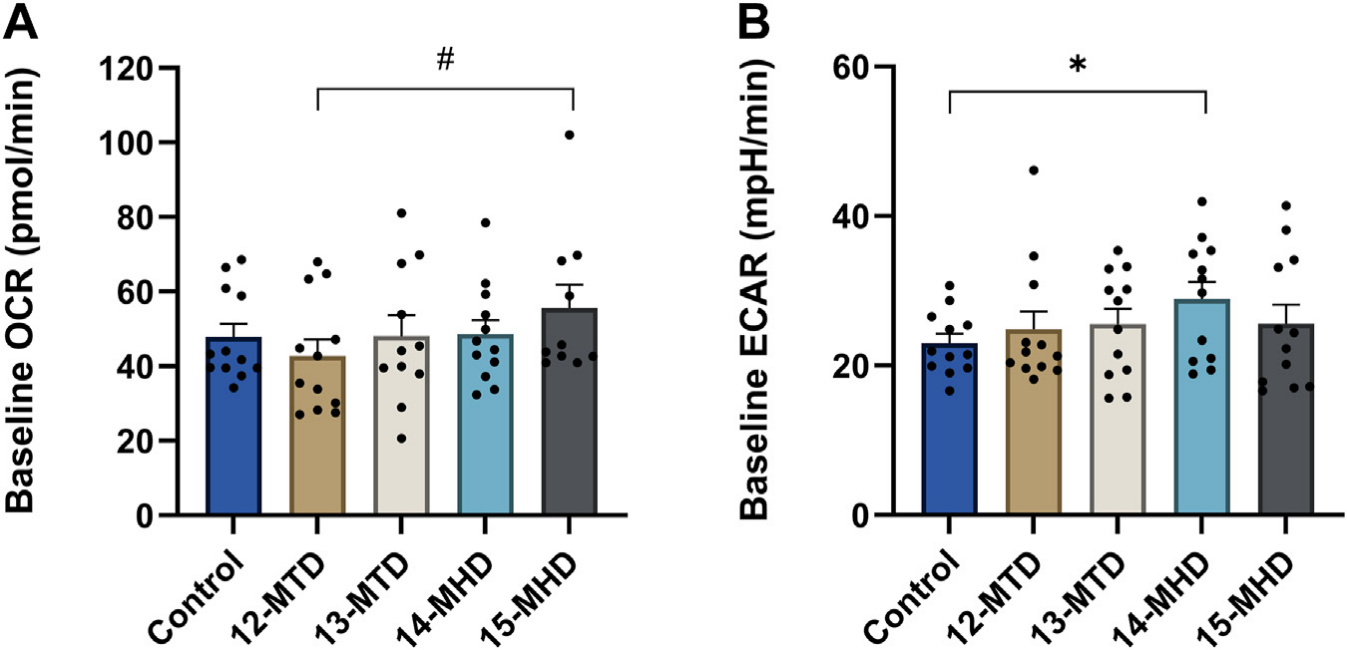
Mitochondrial function in human myotubes after 24 h exposure to branched-chain fatty acids. Human myotubes were grown in 24-well Seahorse tissue culture plates, exposed to 100 μM 12-MTD (12-methyltetradecanoic acid), 13-MTD (13-methyltetradecanoic acid), 14-MHD (14-methylhexadecanoic acid), or 15-MHD (15-methylhexadecanoic acid) for 24 h, before measurement of glycolytic rate with a Seahorse XF24e analyzer. Control myotubes were not exposed to BCFAs. Oxygen consumption rate (OCR) and extracellular acidification rates (ECAR) were recorded six times at 6 min intervals at baseline. A: Mean OCR ± SEM from three individual experiments (n = 3). B: Mean ECAR ± SEM from three individual experiments (n = 3). ∗Significantly increased from control (*P* < 0.05) by paired Student’s *t* test. #Significantly different with the mixed effects model (REML) followed by uncorrected Fisher’s LSD test (*P* < 0.05).

The substrate oxidation experiments above showed that two (13-MTD and 15-MHD) of the four BCFAs examined increased oleic acid oxidation, indicating distinct effects on mitochondrial function. Extracellular flux analyses revealed that 15-MHD increased respiration (OCR) by 30% compared to 12-MTD (Fig. 2A), whereas 14-MHD increased ECAR by 26% compared to control (Fig. 2B). Since mitochondrial manipulations failed to unravel any effects of BCFAs (data not shown), it is unlikely that BCFAs affected mitochondrial biogenesis under the present conditions.

### Effects of BCFAs on glycogen synthesis

**Fig. 3:**
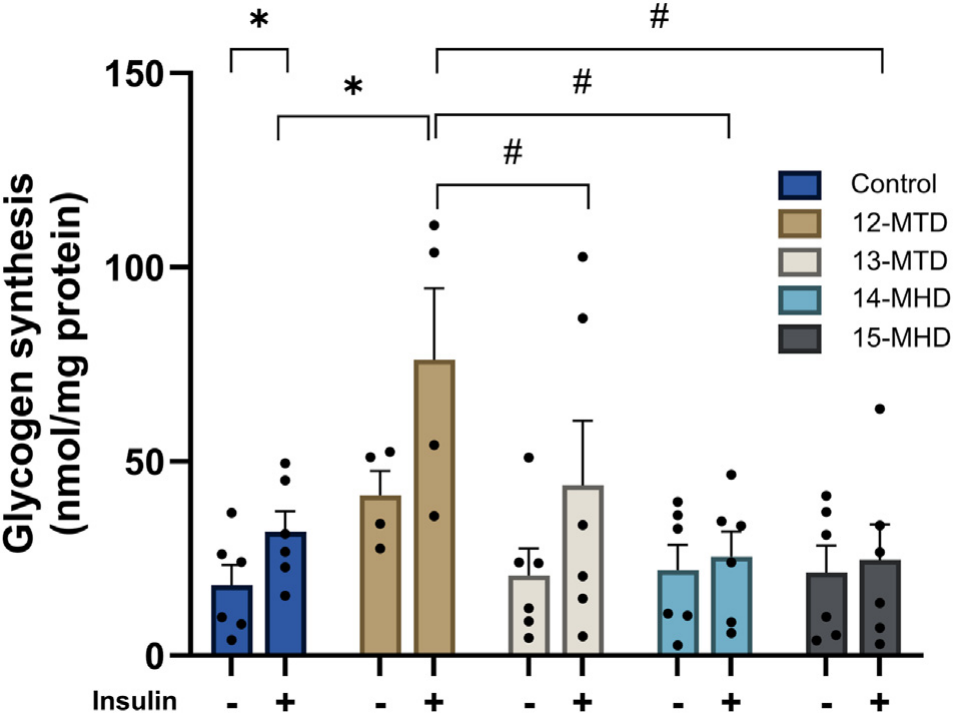
Basal and insulin-stimulated glycogen synthesis in human myotubes treated with branched-chain fatty acids. Human myotubes were incubated with 100 μM 12-MTD (12-methyltetradecanoic acid), 13-MTD (13-methyltetradecanoic acid), 14-MHD (14-methylhexadecanoic acid), or 15-MHD (15-methylhexadecanoic acid) for 24 h. The cells were then incubated for 60 min in serum-free DMEM-Glutamax before D-[^14^C(U)]glucose (1.0 μCi/ml) ± insulin (100 nM) was added, and glycogen synthesis was measured for 2 h. Basal and insulin-stimulated glycogen synthesis given as nmol glycogen/mg cell protein, mean ± SEM from 3 to 6 individual experiments (n = 3–6, each with 2–3 technical replicates). ∗Significantly different from baseline control (*P* < 0.05) by paired Student’s *t* test. #Significantly different with two-way ANOVA followed by uncorrected Fisher’s LSD test (*P* < 0.05).

To investigate possible interaction between BCFAs and insulin, we assessed their separate and synergistic effects on glycogen synthesis (Fig. 3). Insulin-stimulated glycogen synthesis is a sensitive method for assessing insulin responsiveness of human myotubes. We therefore studied the effects of BCFAs on basal and insulin-stimulated glycogen synthesis (Fig. 3). In coherence with increased glucose metabolism, baseline glycogen synthesis seemed to be about 2-fold increased after 24 h exposure to 12-MTD, although not statistically significant (Fig. 3). Interestingly, insulin-stimulated glycogen synthesis increased more than 2-fold in control cells and was further augmented (about 2-fold) by the exposure to 12-MTD. These results suggest that insulin and 12-MTD act independently to increase glycogen synthesis. As for glucose metabolism, 13-MTD, 14-MHD, and 15-MHD did not affect glycogen synthesis per se, and the insulin-stimulated glycogen synthesis was significantly lower after exposure to these BCFAs than to 12-MTD.

### Effects of BCFAs on insulin-stimulated activation of Akt

**Fig. 4:**
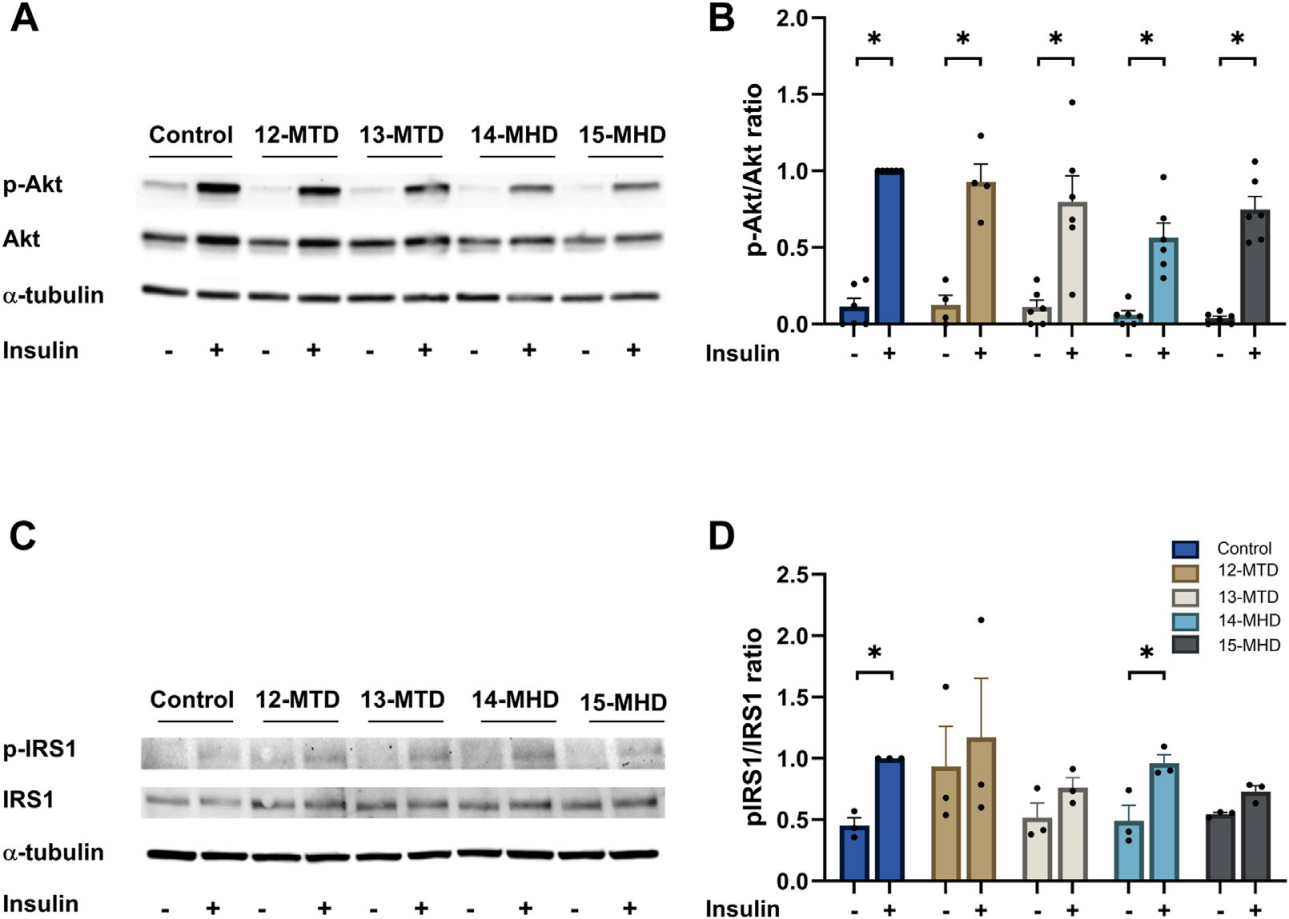
Effect of branched-chain fatty acids on insulin-stimulated phosphorylation of Akt and IRS1. Human myotubes were exposed to 100 μM 12-MTD (12-methyltetradecanoic acid), 13-MTD (13-methyltetradecanoic acid), 14-MHD (14-methylhexadecanoic acid), or 15-MHD (15-methylhexadecanoic acid) for 24 h and then incubated with (+) or without (−) 100 nM insulin for 15 min. Western blot (A) analysis of cell lysates using anti-phospho-Akt (P-Akt), anti-total Akt, and α-tubulin antibodies. A representative blot is shown. Densitometry analysis (B) of P-Akt relative to total Akt. Expression levels were normalized to α-tubulin and related to insulin-treated control. Western blot (C) analysis of cell lysates using anti-phospho-IRS1 (P-IRS1), anti-total IRS1, and a-tubulin antibodies. A representative blot is shown. Densitometry analysis (D) of P-IRS1 relative to total IRS1. Expression levels were normalized to α-tubulin and related to insulin-treated control. Data are given as mean ± SEM from 3–6 individual experiments (B: n = 6 for all treatments except for 12-MTD where n = 4, D: n = 3 for all treatments). ∗Significantly different from insulin-stimulated control (*P* < 0.05) paired Student’s *t* test. #Significantly different with the mixed effects model (REML) followed by uncorrected Fisher’s LSD test (*P* < 0.05).

As the glycogen synthesis experiments above indicated that the different BCFAs have distinct effects on insulin responsiveness, we examined insulin-stimulated phosphorylation of Akt and IRS1 by Western blot analysis (Fig. 4). Insulin stimulated phosphorylation of Akt in all groups of cells (Fig. 4A, B). There was a trend that the same BCFAs which showed reduced insulin-stimulated glycogen synthesis also reduced insulin-stimulated phosphorylation of Akt, although not significantly. For insulin-stimulated tyrosine phosphorylation of IRS1, we observed an about 2-fold increase in control cells and in cells exposed to 14-MHD (Fig. 4C, D), whereas no significant insulin responses were observed after exposure to 12-MTD, 13-MTD, and 15-MHD.

### Impact of BCFAs on protein and lipid synthesis

**Supplemental fig 2:**
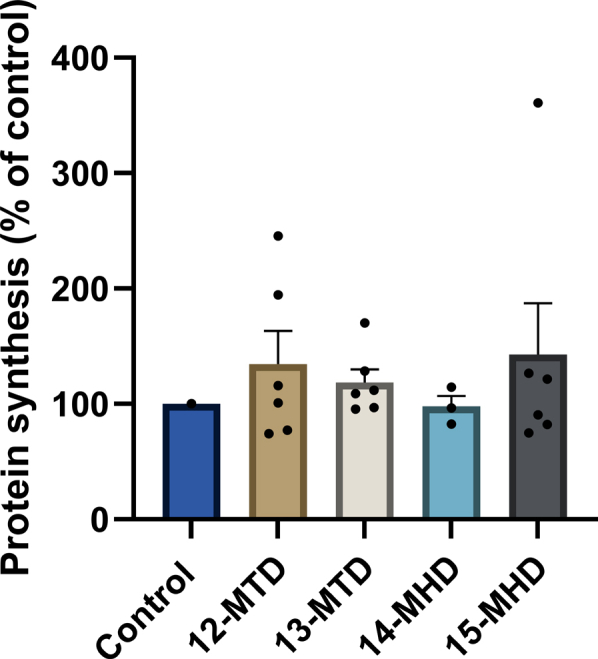


There are some recent reports on increased muscle mass after consumption of dairy products in combination with resistance training (32, 33). To test whether the BCFAs might have a hypertrophic effect on myotubes, protein synthesis was assessed by incorporation of ^14^C-leucine into cellular proteins. No significant effects of BCFAs on ^14^C-leucine incorporation into total cell proteins were observed (supplemental Fig. S2).

**Fig. 5:**
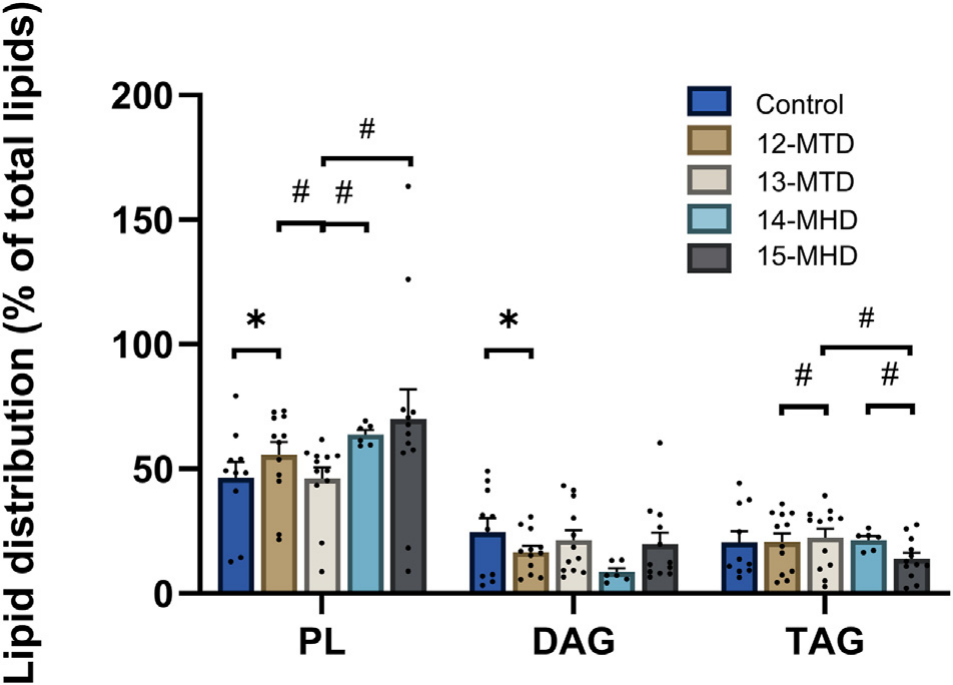
Effect of branched-chain fatty acids on % distribution of ^14^C-leucine into different lipid classes. Human myotubes were exposed to 100 μM 12-MTD (12-methyltetradecanoic acid), 13-MTD (13-methyltetradecanoic acid), 14-MHD (14-methylhexadecanoic acid), or 15-MHD (15-methylhexadecanoic acid) in addition to [^14^C]leucine (1 μCi/ml, 0.8 mM) for 24 h. Control myotubes were not exposed to BCFAs. Cellular lipids were isolated and separated by thin-layer chromatography. The relative distribution into lipid classes was calculated as % of the sum of lipids. Data are given as mean ± SEM from 3 to 6 individual experiments (n = 6 for all treatments except for 14-MTD where n = 3, each with two technical replicates). ∗Significantly different from control (*P* < 0.05) by paired Student’s *t* test. #Significantly different with the mixed effects model (REML) followed by uncorrected Fisher’s LSD test (*P* < 0.05). DAG, diacylglycerol; PL, phospholipids.

To investigate the putative role of the different BCFAs on de novo lipogenesis, incorporation of the ketogenic amino acid ^14^C-leucine into cellular lipids was assessed (Table 2, Fig. 5). Importantly, both total lipid content and the different lipid classes tended to respond distinctively to BCFA treatments, although not all changes reached statistical significance. Total lipid content and the fraction of phospholipids were significantly increased by 13-MTD (Table 2). In addition, 15-MHD increased phospholipid content (Table 2). Assessed as the % of sum of lipids, the fractional distribution into lipid classes, 12-MTD increased the distribution of leucine into phospholipids, whereas the diacylglycerol (DAG) fraction was decreased (Fig. 5). There were also distinct effects of the BCFAs, particularly on phospholipids and triacylglycerols (TAGs) (pairwise comparisons, Fig. 5), and no differences were observed on free fatty acids and cholesterol esters (data not shown).

**Table 2:** Distribution of^14^C-leucin into cellular lipids

	PL	DAG	FFA	TAG	CE	Sum lipids
Control	0.87 (±0.16)	0.37 (±0.09)	0.06 (±0.02)	0.35 (±0.11)	0.05 (±0.01)	1.70 (±0.20)
12-MTD	2.51 (±0.84)	0.65 (±0.20)	0.05 (±0.01)	0.53 (±0.10)	0.11 (±0.03)	3.84 (±1.11)
13-MTD	1.84 (±0.36)^a^	0.87 (±0.29)	0.08 (±0.01)	0.63 (±0.12)	0.08 (±0.01)	3.50 (±0.61)^a^
14-MHD	1.17 (±0.16)	0.17 (±0.05)	0.04 (±0.01)	0.41 (±0.07)	0.06 (±0.02)	1.85 (±0.25)
15-MHD	1.89 (±0.44)^a^	0.46 (±0.13)	0.05 (±0.01)	0.30 (±0.07)	0.08 (±0.02)	2.63 (±0.63)

CE, cholesterol ester; DAG, diacylglycerol; FFA, free fatty acids; PL, phospholipids; TAG, triacylglycerol.

The data are given as average nmol/mg cell proteins (±SEM) (n = 3, each with two technical replicates). Myotubes were incubated with [^14^C]leucine (1 μCi/ml, 0.8 mM) and 100 μM 12-MTD (12-methyltetradecanoic acid), 13-MTD (13-methyltetradecanoic acid), 14-MHD (14-methylhexadecanoic acid), or 15-MHD (15-methylhexadecanoic acid) for 24 h before cellular lipids were extracted and separated by thin layer chromatography.

a Significantly different from control (*P* < 0.05).

### Impact of BCFAs on expression of selected metabolic genes

**Fig. 6:**
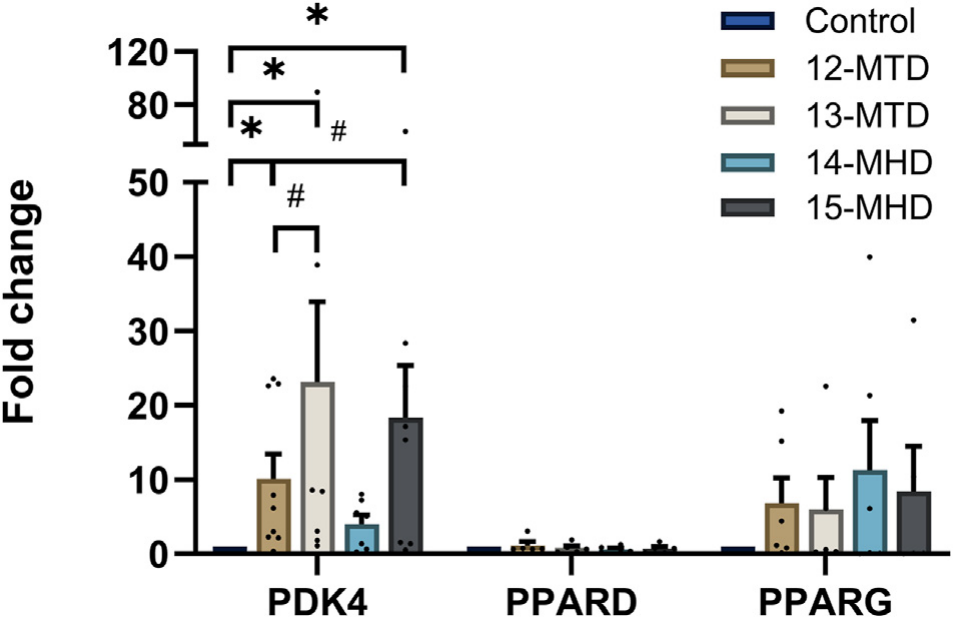
Gene expression in human myotubes after exposure to different branched chain fatty acids. Human myotubes were treated with 100 μM 12-MTD (12-methyltetradecanoic acid), 13-MTD (13-methyltetradecanoic acid), 14-MHD (14-methylhexadecanoic acid), or 15-MHD (15-methylhexadecanoic acid) for 24 h. The cells were harvested for qPCR, and total cell RNA was isolated. The mRNA expressions of *PDK4* (pyruvate dehydrogenase kinase 4), *PPARD* (peroxisome proliferator-activated receptor δ), and *PPARG* (peroxisome proliferator-activated receptor γ) were assessed by RT-qPCR. All values were corrected for the housekeeping control *RPLP0* or *GAPDH* and presented as means ± SEM relative to basal from 3 to 6 individual experiments (n = 3–6, each with two technical replicates). ∗Significantly different from control (*P* < 0.05) by one sample Student’s *t* test. #Significantly different with the mixed effects model (REML) followed by uncorrected Fisher’s LSD test (*P* < 0.05).

**Supplemental fig 3:**
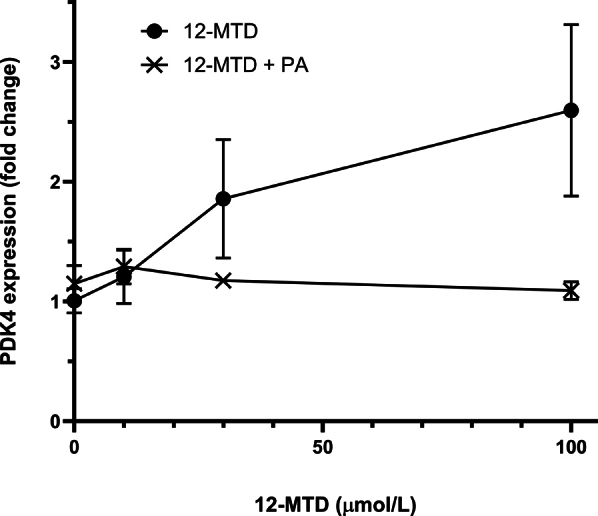


Since all the BCFAs tended to increase fatty acid uptake and oxidation, we examined mRNA expression of selected genes involved in lipid metabolism. Expression of *PDK4* (Fig. 6) was increased by all the tested BCFAs except 14-MHD. 13-MTD and 15-MHD increased *PDK-4* expression significantly more than 12-MTD. We examined the sensitivity of *PDK4* expression to 12-MTD and found a concentration-dependent increase up to 100 μM (supplemental Fig. S3). The mean increase in gene expression of *PPAR-γ* also tended to increase (6-fold to 11-fold) by all the BCFAs, but not significantly.

The observed effects of 12-MTD on glucose metabolism and glycogen synthesis support a distinct role compared to the other BCFAs. However, we did not observe any effect of BCFAs on gene expression of the glucose transporters *GLUT1* or *GLUT4* (supplemental Fig. S4). The effects of 13-MTD, 14-MHD, and 15-MHD on oleic acid uptake and oxidation were neither accompanied by increased mRNA expression of the fatty acid transporter *CD36* nor increased expression of the transporter of fatty acids into mitochondria (*CPT-1B*) or the mitochondrial enzyme *CYC1* (supplemental Fig. S4).

## Discussion

In the present study, we examined the effect of BCFAs on energy metabolism in human myotubes. The myotubes were exposed to different BCFAs for 24 h prior to measuring both catabolic and anabolic reactions. The findings show that the different BCFAs have distinct effects on the measured biochemical pathways. 12-MTD increased glucose metabolism, i.e., glucose uptake and glycogen synthesis, whereas 13-MTD, 14-MHD, and 15-MHD improved oleic acid metabolism. Additionally, the study showed that the *iso*-BCFAs 13-MTD and 15-MHD seemed to have quite similar effects, such as increasing oleic acid metabolism, increasing the phospholipid fraction of cellular lipids, and enhancing mRNA expression of *PDK4*. The *anteiso*-BCFAs 12-MTD and 14-MHD had more diverse effects, which were both similar as well as divergent.

This study mainly aimed at elucidating the impact of BCFAs on glucose and fatty acid (oleic acid) metabolism in human myotubes. The *anteiso*-BCFA 12-MTD increased anabolic glucose pathways, such as glucose uptake and glycogen synthesis, whereas 13-MTD, 14-MHD, and 15-MHD did not significantly affect glucose metabolism. Oleic acid uptake was increased by 13-MTD, 14-MHD, and 15-MHD and oleic acid oxidation by 13-MTD and 15-MHD. The results from the mitochondrial function assay were, however, conflicting, showing increased glycolytic flux by 14-MHD and as shown in the substrate oxidation assay, enhanced mitochondrial function by 15-MHD, but not by 13-MTD and 14-MHD. When BCFAs were added in combination with palmitic acid (supplemental Fig. S1), the effects of BCFAs were masked, implying that there is a competition between branched and nonbranched fatty acids and that a high concentration of a saturated fatty acid can combat the metabolic effects of BCFAs. Taken together, these findings show metabolic effects of BCFAs on human myotubes and suggest that the different BCFAs have divergent effects on energy substrate uptake and oxidation in skeletal muscle cells, indicating an effect of the *anteiso*-forms mainly on glucose metabolism and the *iso*-forms on oleic acid metabolism.

To the best of our knowledge, effects of BCFAs on myotubes have not previously been shown. Clinical studies, however, have shown that obesity is associated with low levels of BCFAs in plasma (18) and adipose tissue (34). An increased fraction of BCFAs in adipose tissue was seen after weight loss by bariatric surgery, and relative content of BCFAs in the adipose tissue correlated positively with insulin sensitivity (34). Recently, circulating BCFAs were also found to associate with insulin sensitivity and beta cell function in a longitudinal study including subjects with type 2 diabetes (35). In particular, *iso*-15:0 (13-MTD) and *anteiso*-15:0 (12-MTD) were positively associated with insulin sensitivity. Yehia *et al.* (35) points to a link between tetradecanoic BCFAs and glucose metabolism, which, in line with these data, alludes to a link between distinct BCFAs and skeletal muscle energy metabolism.

The effects of BCFAs on insulin responsiveness were also diverse in our study, assessed by insulin-stimulated glycogen synthesis and insulin-stimulated phosphorylation of Akt and IRS1. None of the studied BCFAs seemed to enhance insulin responses, although such effects might have been difficult to see due to the supraphysiological concentration of insulin (100 nM). In contrast, 13-MTD, 14-MHD, and 15-MHD suppressed insulin-stimulated glycogen synthesis, and 12-MTD, 13-MTD, and 15-MHD blunted insulin-stimulated tyrosine phosphorylation of IRS1. However, insulin-stimulated phosphorylation of Akt was not affected by any of the BCFAs. These results on insulin sensitivity were not very conclusive and contradictory to a previous in vivo observation that showed a positive correlation between adipose tissue BCFA content and insulin sensitivity (34). However, our results are in accordance with the recent observation by Yehia *et al.*, who showed that the association between different BCFAs and insulin sensitivity was fatty acid specific (35).

As seen above (Fig. 3), 12-MTD increased the anabolic incorporation of glucose into glycogen. We also wanted to study the effect of the different BCFAs on other anabolic reactions, such as protein synthesis, as it has been suggested that dairy products can enhance the hypertrophic effect of resistance training (32, 33). However, we did not observe any effect of the BCFAs on leucine incorporation into cellular proteins in myotubes after pretreatment for 24 h, implying no isolated effect of the BCFAs on protein synthesis. However, any effect on protein synthesis in combination with contractile activity, such as electrical pulse stimulation, is possible and would have been interesting to study.

It has been shown that 12-MTD is able to induce expression of lipid synthesis genes (*FASN, ELOVL4,* and *ELOVL6*) in human adipocytes (11), and the increased expression of *FASN* by 12-MTD was recently also shown in hepatocytes (36). Thus, we studied the impact of BCFAs exposure on de novo lipogenesis in human myotubes with leucine as the precursor substrate. 12-MTD, 13-MTD, and 15-MHD seemed to increase leucin incorporation into total lipid content, although only the effect of 13-MTD was significant, whereas both 13-MTD and 15-MHD increased the content of leucine in the phospholipid fraction. The relative distribution of newly synthesized lipids was changed by 12-MTD, with an increase in the phospholipid fraction and a decrease in the DAG fraction. These results demonstrate that the BCFAs modify lipid synthesis and storage in skeletal muscle cells, and we know from previous studies that TAG content of skeletal muscles is highly connected to insulin sensitivity and metabolic flexibility (37). An increased DAG content is particularly linked to insulin resistance through activation of protein kinase C (38). A relative reduction of DAG after exposure to 12-MTD might therefore be noteworthy. The TAG content was, however, unchanged by all the examined BCFAs (Table 2). However, it must be emphasized that de novo lipogenesis is generally very low in human myotubes (31).

The mechanism by which BCFAs affect energy metabolism in myotubes is unknown. To elucidate possible mechanisms, we studied expression of selected metabolic genes. The increased glucose uptake by 12-MTD could not be explained by increased *GLUT1* gene expression, neither could the increased oleic acid uptake by 13-MTD, 14-MHD, and 15-MHD be explained by increased expression of the fatty acid transporter *CD36*. Expression of *GLUT4* was not affected by any of the BCFAs, and thus the increased insulin-stimulated glycogen synthesis by 12-MT could not be explained by increased insulin-stimulated glucose uptake. This is as expected since expression of *GLUT4* is known to be low in these cells (39). The effects of 13-MTD and 15-MHD on oleic acid oxidation could not be accounted for by changes in the mitochondrial genes *CPT1B* and *CYC1*. However, except for 14-MHD, *PDK4* mRNA expression was increased by all the BCFAs. Among them, 13-MTD and 15-MHD showed the greatest effects, suggesting that the BCFAs might act as PPAR-δ-agonists (40). There were also significant differences in the *PDK4* mRNA expression levels between the BCFAs, where both the effect of 13-MTD and 15-MHD was greater than the effect of 12-MTD. This observation is in accordance with 13-MTD and 15-MHD being better stimulators of oleic acid oxidation (41). Expression of *PPAR-γ* also tended to be increased by all the examined BCFA, although not significantly. Some BCFAs have previously been found to activate PPAR-α in rat hepatoma cells; however, different BCFAs were used compared to our study (42). PDK4 is known as a regulator of metabolic flexibility. By phosphorylating and inhibiting pyruvate dehydrogenase (PDH), PDK4 shifts cellular metabolism to favor fatty acid oxidation over glucose oxidation (43). Thus, an increased expression of *PDK4* might explain the increased oleic acid oxidation seen by the BCFAs. Further work is needed to determine whether the effect is mediated through PPARs or alternatively may be ascribed to, e.g., allosteric effects caused by slightly different degradation pathway of the branched versus nonbranched saturated fatty acids, as indicated by the nullifying effect of palmitic acid (supplemental Fig. S1).

BCFAs have been proposed to have a favorable cardiometabolic role, because of an inverse association between plasma levels of BCFAs and obesity, insulin resistance, and inflammatory markers (10). It has been shown that lipid oxidation is reduced in skeletal muscle from obese individuals (44, 45). Interestingly, Mika *et al.* have shown that lean subjects have higher levels of *iso*-BCFAs in serum than obese individuals (18). In our study, both *iso*-BCFAs, 13-MTD and 15-MHD, increased oleic acid oxidation compared to the control. Even though our study was performed on skeletal muscle cells in culture, these findings suggest a potential role for *iso*-BCFAs in enhancing lipid oxidation, which may be impaired in obese individuals.

Overall, the present study sheds light on the mechanisms by which BCFAs affect energy metabolism in skeletal muscle cells and points to potential therapeutic applications of BCFAs in improving energy metabolism. However, further research is needed to fully understand the effects of BCFAs on energy metabolism and their long-term safety and efficacy. The present study has some limitations that should be considered when interpreting the results. The study was conducted in vitro using cultured human myotubes, which may not fully reflect the metabolic environment of living organisms. The metabolic effects of BCFAs in vivo may differ from those observed in this study, and further research is needed to validate the findings in animal models and human subjects. The study also used relatively high concentrations of BCFAs, which may not be physiologically relevant, and a fixed time of exposure that might not be optimal for all BCFA-mediated effects.

The findings of this study suggest that different BCFAs have distinct effects on fatty acid and glucose metabolism, as well as mitochondrial function of skeletal muscle cells. This suggests that specific BCFAs could be targeted to modulate metabolic pathways in a manner that is beneficial for metabolic health. For example, 12-MTD could be targeted to improve glucose metabolism in skeletal muscle, whereas 13-MTD, 14-MHD, and 15-MHD could be targeted to improve fatty acid uptake and oxidation and mitochondrial function. Furthermore, the study found that the BCFAs had differential effects on insulin sensitivity, which should be considered in a potential therapeutic application.

## Data availability

The data that support the findings of this study are available from the corresponding author upon reasonable request.

## Supplemental data

This article contains supplemental data.

## Conflict of interest

The authors declare that they have no conflicts of interest with the contents of this article.

## Figures and Tables

**Supplemental fig 4:**