# Supplementary material for: Divergent effects of monomethyl branched-chain fatty acids on energy metabolism and insulin signaling in human myotubes
Source: J Lipid Res. 2025 Feb 24;66(3):100764. doi: 10.1016/j.jlr.2025.100764 (PMC11982973; doi:10.1016/j.jlr.2025.100764)
Supplement: Supplemental fig legends [file mmc1.docx]

**Supplementary figure legends**

**Supplementary figure 1:** Glucose and oleic acid uptake and oxidation in human myotubes treated with branched-chain fatty acids, palmitic acid, or in combination. Human myotubes were incubated with 100 µM 12-MTD (12-methyltetradecanoic acid), 13-MTD (13-methyltetradecanoic acid), 14-MHD (14-methylhexadecanoic acid) or 15-MHD (15-methylhexadecanoic acid) in combination with (+) or without (-) 300 µM palmitic acid (PA) for 24 h before glucose (A, B) and oleic acid (C, D) metabolism were assessed by addition of a medium containing [^14^C]glucose (0.5 ​μCi/ml, 200 ​μM) or [^14^C]oleic acid (0.5 ​μCi/ml, 100 ​μM) for 4 h. Cell-associated radioactivity and trapped CO_2_ (oxidation) were measured as described in the methods. Uptake of glucose and oleic acid were calculated as the sum of oxidized substrate and cell-associated radioactivity. Data are given as mean ± SEM from 6 individual experiments (n=6, each with 4 technical replicates). In control cells, glucose uptake and oxidation were 85.6 ± 44.5 nmol/mg and 78.9 ± 42.8 nmol/mg, respectively, and increased to 101.7 ± 50.4 nmol/mg and 95.3 ± 49.1 nmol/mg, respectively after exposure to PA. Oleic acid uptake and oxidation were 14.8 ± 6.0 nmol/mg and 0.9 ± 0.1 nmol/mg, respectively, and increased to 16.5 ± 6.5 nmol/mg and 1.3 ± 0.2 nmol/mg, respectively after exposure to PA. *Significantly different from control (p<0.05) by one sample t-test.

**Supplementary figure 2.** Effect of branched chain fatty acids on protein synthesis. Human myotubes were exposed to 100 µM 12-MTD (12-methyltetradecanoic acid), 13-MTD (13-methyltetradecanoic acid), 14-MHD (14-methylhexadecanoic acid) or 15-MHD (15-methylhexadecanoic acid) in addition to [^14^C]leucine (1 µCi/ml, 0.8 mM) for 24 h. Control myotubes were not exposed to BCFA. The cell proteins were precipitated and incorporation of ^14^C-leucine into cellular proteins were assessed by liquid scintillation counting. Data are given as mean ± SEM from 3-6 individual experiments (n=6 for all treatments except for 14-MTD where n=3, each with 3-4 technical replicates).

**Supplementary figure 3.** Gene expression of *PDK4* in human myotubes after exposure to different concentrations of 12-MTD (12-methyltetradecanoic acid). The cells were treated with 10, 30 or 100 µM 12-MTD for 24 hours, harvested for qPCR and total cell RNA was isolated. The mRNA expression of *PDK4* (pyruvate dehydrogenase kinase 4) was assessed by RT-qPCR. The values were corrected for the housekeeping control *GAPDH* and presented as means ± SEM from 2 individual experiments relative to basal (n=2, each with 3 technical replicates).

**Supplementary figure 4.** Gene expression in human myotubes after exposure to different branched chain fatty acids. Human myotubes were treated with 100 µM 12-MTD (12-methyltetradecanoic acid), 13-MTD (13-methyltetradecanoic acid), 14-MHD (14-methylhexadecanoic acid), or 15-MHD (15-methylhexadecanoic acid) for 24 h. The cells were harvested for qPCR and total cell RNA was isolated. The mRNA expressions of *CD36* (fatty acid transporter), *GLUT1* (glucose transporter 1), *CYC1* (mitochondrial cytochrome c1), and *CPT1B* (carnitine palmitoyl-transferase 1B) were assessed by RT-qPCR. All values were corrected for the housekeeping control RPLP0 and presented as means ± SEM from 3-9 individual experiments relative to basal (n=9 for *CD36* and *GLUT1*, n=3 for *GLUT4*, n=6 for *CPT1B* and *CYC1*, each experiment with 2 technical replicates).
